# The influence of physical activity, adherence to Mediterranean diet, and weight status on the psychological well-being of adolescents

**DOI:** 10.1186/s40359-024-01906-3

**Published:** 2024-07-27

**Authors:** Adrián Mateo-Orcajada, Lucía Abenza-Cano, Juan Manuel Molina-Morote, Raquel Vaquero-Cristóbal

**Affiliations:** 1 Facultad de Deporte. UCAM, Universidad Católica de Murcia, Murcia, Spain; 2 Research Group Movement Sciences and Sport (MS&SPORT), Department of Physical Activity and Sport, Faculty of Sport Sciences, University of Murcia, Murcia, Spain

**Keywords:** Basic psychological needs, Life satisfaction, Physical activity level, Youth

## Abstract

The mental health of adolescents is a determining factor for their adequate development, but is influenced by factors such as physical activity, nutrition, gender, and weight status. However, previous research has not analysed differences in psychological status, mainly in basic psychological needs and life satisfaction, among male and female adolescents with different levels of physical activity, weight status and adherence to the Mediterranean diet (AMD). For this reason, the objectives of the present investigation were to establish whether the differences between active and inactive adolescents in basic psychological needs and life satisfaction depend on gender; and to determine the differences in basic psychological needs and life satisfaction of active and inactive adolescents with different weight status and AMD. A total of 791 adolescents aged between twelve and sixteen years old participated in the study. All the participants were measured for basic psychological needs, life satisfaction, and level of physical activity, AMD, and height and body mass. The results showed a higher score in basic psychological needs and life satisfaction for active adolescents in both the males’ and females’ groups. No differences were found in the psychological variables when comparing adolescents with different weight status. Adolescents with a higher AMD showed higher scores in satisfaction of basic psychological needs and satisfaction with life than adolescents with a worse AMD. Therefore, it can be concluded that the level of physical activity and AMD are factors to be considered for the mental health of adolescents, but the relevance of weight status will have to be confirmed in future research.

## Introduction

Adolescence is a complicated stage in which physical and psychological changes occur [1]. At this stage, the subject leaves childhood behind and prepares for adulthood, leaving the adolescent in a state of psychological, emotional, and social vulnerability [2]. It is a stage of great risks and opportunities, as most of the habits adopted at this stage will have an impact on lifestyle and future health [3]. In this sense, a relevant change during this stage is the decrease of the practice of physical activity among adolescents [4]. This is due to factors such as increased academic demands, lack of time, lack of interest in sport [5], or other leisure time preferences [6–8]. This has negative physical, psychological and body composition consequences for adolescents [9, 10].

Body composition also plays a very important role at this stage as the changes that occur, mainly weight gain and changes in body composition characterized by an increase in both muscle mass and fat mass [11, 12]. These changes are the origin of numerous problems related to body image satisfaction [13], especially among women [13], which negatively affects the psychological state of this population [14]. Added to this is the fact that physical activity decreases during this stage [15], which increases fat accumulation in this population [16], and could aggravate problems related to body image [19]. Because of all the above, adolescence is a particularly sensitive stage for the appearance of nutritional problems and eating disorders [13]. This is because changes in nutritional habits are sought to compensate for the changes that are taking place in the body [13], influencing the type of food eaten as well as the quantities [13, 17]. This could seriously affect the physical and psychological health of adolescents as adolescence is a nutrition-sensitive phase for growth [18] and it is also a habit-forming stage [19, 20].

Therefore, as has been shown in the literature, the close relationship between physical activity, weight status and nutritional patterns means that changes in any of these aspects influence the physical and psychological health of adolescents [21–23]. Specifically, in the psychological domain, it is basic psychological needs and life satisfaction that seem to be significantly affected by these changes, which is very relevant because of their relation to well-being [24]. Thus, adolescents with a lower level of physical activity and adherence to the Mediterranean diet (AMD), which is one of the most complete nutritional patterns [25], show lower satisfaction of basic psychological needs [26, 27]. In the case of weight status, it has been observed that adolescents with obesity, who generally report lower frequency and intensity of physical activity, have lower satisfaction of basic psychological needs [28].

In addition, it should be noted that these effects do not occur equally in adolescent males and females [29, 30]. In general, this is due to the differences found during adolescence in the practice of physical activity, with males being more active during adolescence [15, 31]; in AMD, with females tending to be more adherent [30]; and in fat mass accumulation, with females accumulating more adiposity due to hormonal changes [12]. However, no previous research is known that has analysed the joint influence of healthy habits on the psychological state of adolescents, and whether this influence differs according to gender, so it is not known whether active and inactive male and female adolescents with different adherence to a nutritional pattern or weight status have differences in their mental health. For this reason, the aims of the present research were (a) to establish whether the differences between active and inactive adolescents in basic psychological needs and life satisfaction depend on gender; (b) to determine the differences in basic psychological needs and life satisfaction of active and inactive adolescents with different weight status; and (c) to analyse the differences in basic psychological needs and life satisfaction of active and inactive adolescents with different AMD.

## Methods and materials

Following the STROBE statement [32], a cross-sectional research design was carried out. The research and measurement protocol were reviewed and approved by the institutional ethics committee of the Catholic University of Murcia before the start of the study (code: CE022102), following the indications of the World Medical Association and the Helsinki declaration. Four public schools in different geographical areas of the Region of Murcia (Spain), selected because they had the largest number of students in mandatory education in their localities, participated in the study. The study took place in the academic years 2020/2021 and 2021/2022.

### Participants

A total of 791 adolescents, 404 males and 387 females between the ages of twelve and sixteen years old (mean age: 14.39±1.26 years old) and generally with medium socio-economic status finally participated in the research. All participants were in compulsory secondary education, which is the first to fourth grade after primary school (1st : 206 adolescents, 110 males, 96 females; 2nd : 133 adolescents, 64 males, 69 females; 3rd : 238 adolescents, 124 males, 114 females; and 4th : 214 adolescents, 106 males, 108 females). The mean physical activity score for the sample was 2.64±0.67 score; the mean body mass index (BMI) was 21.35±3.96 kg/m^2^; and the mean AMD was 6.48±2.48 score. A total of 352 adolescents were active and 438 inactive; 231 were overweight/obese and 560 normal weight; and 91 have poor AMD, 418 need to improve AMD and 282 have optimal AMD. Regarding the disease history, in general the adolescents showed no difficulties in functionality (1.16±0.08 score from 1 to 5 scale), nor in the degree of discomfort (1.27±0.13 score from 1 to 5 scale), and some reported occasional pain in the lumbar region (21.96%), neck (24.36%) and shoulders (7.81%).

All the participants gave their consent to voluntarily participate in the research before the beginning of the study, and their parents completed the informed consent form authorizing them to participate. The sample selection was non-probabilistically by convenience. Rstudio software (3.15.0 version, Rstudio Inc., Boston) was used to perform the sample size calculations, using the standard deviations (SD) from previous research that examined life satisfaction (SD = 0.78) in adolescents aged twelve to sixteen years old. With an estimated error (d) of 0.07 for life satisfaction, for a 99% confidence interval, the sample size needed was 756 adolescents. The significance level for the calculation was set as α = 0.05.

All the participants met the following inclusion criteria: (a) attending compulsory secondary education; (b) aged between twelve and sixteen years old; (c) not presenting any mental illness that would impede the understanding of the questionnaires; (d) not having suffered an operation, illness, or injury that would have prevented normal physical activity in the past week; (e) not having any metabolic or autoimmune disease that would lead to changes in their eating habits; and (f) completing all the questionnaires in their entirety.

### Instruments

#### Questionnaire measurements

An ad hoc questionnaire was designed to collect socio-demographic information about their gender, age, academic year, and disease history. The questions used to obtain information on disease history were based on a questionnaire previously validated, the “Short Musculoskeletal Function Assessment Questionnaire” (SMFA) [33], which was used to determine upper and lower extremity dysfunctionalities [33, 34], as this information, together with the presence of metabolic and autoimmune diseases, were used as exclusion criteria for the present investigation. This questionnaire was used in its Spanish version, which also had previous validation. This questionnaire includes 46 questions (34 covering the assessment of the patients function and 12 covering how bothered patients are by their symptoms). A 5-point Likert scale (from 1: no problems/no difficulty/not bothered; to 5: unable to do/symptoms all the time/being greatly bothered) is used for completion. The arithmetic means of the sum of the questions in both functionality and discomfort categories is used as the final score of the category, ranging from 1 to 5 points. A higher score on this scale shows greater problems of functionality and discomfort. For the present research, the SMFA showed a Cronbach’s alpha of 0.773.

The psychological variables were recorded using the Satisfaction with Life Scale (SWLS) [35] and the Basic Psychological Needs Scale (BPNS) [36]. Both scales were originally created and validated in English and then translated and validated in Spanish, which is the version used for the present research for SWLS [37] and BPNS [38]. The SWLS is composed of 5 items. This scale is completed on a 5-points Likert scale (from 1: strongly disagree; to 5: strongly agree). The final score is obtained by adding up the scores of the five items, so the scores ranges between 5 and 25 points, with a higher score indicating greater satisfaction with life. The internal consistency shown by this scale in previous research was high (α = 0.84) [39], as well as in the results found for the sample of adolescents in the present study (α = 0.800). About the BPNS is composed of three dimensions: competence, autonomy, and relatedness. The total scale is composed of 18 items, 6 belonging to each dimension. It is completed on a 6-points Likert scale (from 1: totally false; to 6: totally true). The final score is obtained by adding up the scores of the six items of each category, so the final score for each category ranges from 6 to 36 points, with a higher score meaning greater satisfaction of the basic psychological need. The psychometric analyses conducted in previous research showed the adequate internal consistency of BPNS scale (competence = 0.80; autonomy = 0.69; and relatedness = 0.73) for use with adolescents [40]. For the present research, this scale showed a high internal consistency (α = 0.896 for the whole scale; competence: 0.911; autonomy: 0.802; relatedness: 0.733).

The Physical Activity Questionnaire for Adolescents (PAQ-A) was used to determine the level of physical activity of adolescents. This questionnaire was originally created and validated in English [41, 42]; and later translated and validated in Spanish, which was the version used in the current research [42]. This questionnaire is composed of a total of nine items. The first eight items are completed on a 5-points Likert scale (from 1: no physical activity; to 5: a lot of physical activity), while the ninth item is completed dichotomously (yes or no). The arithmetic mean of the scores of the first eight questions was used to establish the final physical activity score. The final score of the questionnaire ranges from 1 to 5 points. The ninth item allow to find out whether in the last week the adolescent had any problem that prevented him/her from engaging in normal physical activity. In order to classify adolescents as active or sedentary, a cut-off point of 2.75 was established, with active adolescents being those who obtained this score or higher, and sedentary adolescents being those who obtained a lower score [43]. This questionnaire had an intraclass correlation coefficient of 0.71 for the final score of the questionnaire, making it a valid and reliable instrument for assessing the physical activity of adolescents [42]. For the present research, the PAQ-A showed a Cronbach’s alpha of 0.859.

The Mediterranean Diet Quality Index for children and teenagers (KIDMED) questionnaire [44] was selected to determine the AMD of the adolescents. This questionnaire was originally created and validated in Spanish, which is the version used in this research [37]. This questionnaire is composed of 16 items in which adolescents must indicate compliance or non-compliance with each item (dichotomous answer: yes or no). The response “yes” to each question gives a score of + 1 (positive connotation) or -1 (negative connotation). A total of 12 items have a positive connotation and 4 negative. The final score is the sum of the items, so the final score ranges from 0 to 12 points. The final score allows three categories to be established: low AMD (0–3 points), medium AMD (4–7 points), or optimal AMD (8–12 points) [44]. This questionnaire showed moderate reliability and reproducibility values in previous research (α = 0.79) [45]. For the present research, the PAQ-A showed a Cronbach’s alpha of 0.778.

#### Weight status measurement

All kinanthropometric measurement were performed according to the protocol standardized by the International Society for the Advancement of Kinanthropometry (ISAK) [46].

To determine the weight status, one accredited ISAK anthropometrist (level 2) measured body mass and height of the adolescents, using a TANITA BC 418-MA Segmental scale (TANITA, Tokyo) with an accuracy of 100 g, and a SECA stadiometer 213 (SECA, Hamburg) with an accuracy of 0.1 cm., respectively. Both variables were taken twice, with a third measurement performed when the difference between the first and second measurements was greater than 1%. The mean between measurements, in the case of two attempts, and the median, in the case of three attempts, was taken as the final value. The intra-evaluator technical error of measurements (TEM) was calculated in a sub-sample and a value of 0.02% was obtained.

The final values of body mass and height were used to calculate BMI (body mass / height^2^). According to the World Health Organization, in the present study, the weight status of adolescents was established as normal weight when the BMI value was similar or lower than 24.9, and overweight/obese when the BMI value was similar or higher than 25. No differentiation was made between overweight and obese adolescents because the sample size of the separate groups was too small.

### Procedure

All measurements were carried out on the same day, taking advantage of the physical education class time in the participating secondary schools. All the adolescents performed the tests under the same conditions, using the covered pavilion of each school, thus eliminating the contaminating variables of the environment as much as possible.

First of all, the protocol included the self-completion of the ad hoc questionnaire based on SMFA, to determine upper and lower extremity dysfunctionalities and the presence of metabolic and autoimmune diseases. After that, the PAQ-A, KIDMED, BPNS and SWLS questionnaires were completed in a random order, with the adolescents seated and in a calm environment, without noise or distractions that could influence their responses. The researchers only resolved the adolescents’ doubts but did not help them to complete the questionnaire to avoid conditioning their responses. Finally, height and body mass were measured.

### Data analysis

The Kolmogorov-Smirnov normality test was used to determine the distribution of the data. All the variables followed a normal distribution, so the statistical analysis was composed of parametric tests. Descriptive statistics were used to find the mean values and standard deviations. A one-factor ANCOVA was performed to analyze the differences in basic psychological needs and life satisfaction between physically active and sedentary adolescents, with gender, quality of diet, and weight status as covariates in the model. Three MANOVA analyses were performed, the first to establish the differences between active and inactive males and females, the second to determine the differences between active and inactive adolescents according to whether they were overweight/obese or normal weight, and the third to analyze the differences between active and inactive adolescents with different quality diets. Bonferroni’s pairwise comparison was used for variables that were statistically significant. Partial eta squared (η2) was used to calculate the effect size and was defined as small: ES ≥ 0.10; moderate: ES ≥ 0.30; large: ≥ 1.2; or very large: ES ≥ 2.0, with an error of *p* < 0.05 [47]. A value of *p* < 0.05 was set to determine statistical significance. The statistical analysis was performed with the SPSS statistical package (v. 25.0; SPSS Inc., IL).

## Results

Table 1 shows the differences between active and inactive adolescents in their basic psychological needs and life satisfaction. The results showed that active adolescents presented higher scores than inactive adolescents in all the variables analyzed (*p* < 0.001). The covariates included showed influence on all the variables analysed: competence (gender: *p* < 0.001; diet quality: *p* < 0.001; weight status: *p* < 0.001), autonomy (gender: *p* < 0.001; diet quality: *p* < 0.001; weight status: *p* < 0.001), relatedness (gender: *p* < 0.001; diet quality: *p* = 0.001; weight status: *p* < 0.001), and life satisfaction (gender: *p* = 0.002; diet quality: *p* = 0.002; weight status: *p* < 0.001). The effect size was moderate for all variables analysed, as well as when considering the influence of covariates on the differences found. However, the effect size was higher for the competence variable than for the other variables.

**Table 1:** Differences in basic psychological needs and life satisfaction between active (*n* = 352) and inactive (*n* = 438) adolescents according to gender, diet quality and obesity level

Descriptors							Gender covariate				Diet Quality covariate				Weight Status covariate		
	Active	Inactive	F	*p*	ES (η2)		F	*p*	ES (η2)		F	*p*	ES (η2)		F	*p*	ES (η2)
Competence	28.96±6.67	24.50±7.62	74.755	< 0.001	0.870		59.583	< 0.001	0.700		61.616	< 0.001	0.730		84.271	< 0.001	0.970
Autonomy	26.68±6.47	24.33±6.78	24.494	< 0.001	0.300		19.955	< 0.001	0.250		17.256	< 0.001	0.210		28.698	< 0.001	0.350
Relatedness	25.55±6.28	23.57±6.63	18.312	< 0.001	0.230		15.449	< 0.001	0.190		11.783	0.001	0.150		22.772	< 0.001	0.280
Life satisfaction	18.46±4.56	17.17±4.95	14.235	< 0.001	0.180		10.066	0.002	0.130		9.806	0.002	0.120		16.977	< 0.001	0.210

ES: effect size

Regarding the differences between active and inactive males, and active and inactive females, it should be noted that the basic psychological needs was higher in active adolescents, both in the group of males (*p* < 0.001–0.012) and in the group of females (*p* < 0.001–0.002), than sedentary adolescents, while for life satisfaction, only active females showed a significantly higher value than inactive females (*p* < 0.001) (Table 2). The effect size was moderate on competence and autonomy for males and females. However, in relatedness, the effect size was moderate for females, but small for males; while in life satisfaction, it was small for females and moderate for males.

**Table 2:** Differences in basic psychological needs and life satisfaction in adolescents’ males (*n* = 403) and females (*n* = 387) according to their physical activity level

	Gender	Active	Inactive	Mean. Diff.	95% CI Diff.	*p*	ES (η2)
Competence	Males	29.12±6.68	25.75±7.56	3.366	1.947; 4.785	< 0.001	0.270
	Females	28.66±6.67	23.68±7.57	4.988	3.446; 6.531	< 0.001	0.490
Autonomy	Males	26.84±6.42	24.75±6.80	2.092	0.780; 3.404	0.002	0.120
	Females	26.37±6.57	24.05±6.76	2.320	0.893; 3.746	0.001	0.130
Relatedness	Males	25.58±6.00	23.94±6.84	1.646	0.366; 2.926	0.012	0.080
	Females	25.50±6.81	23.33±6.48	2.168	0.776; 3.559	0.002	0.120
Life satisfaction	Males	18.43±4.33	17.91±4.70	0.513	-0.429; 1.454	0.285	0.160
	Females	18.52±4.99	16.68±5.05	1.842	0.818; 2.865	< 0.001	0.010

ES: effect size.

**Fig. 1:**
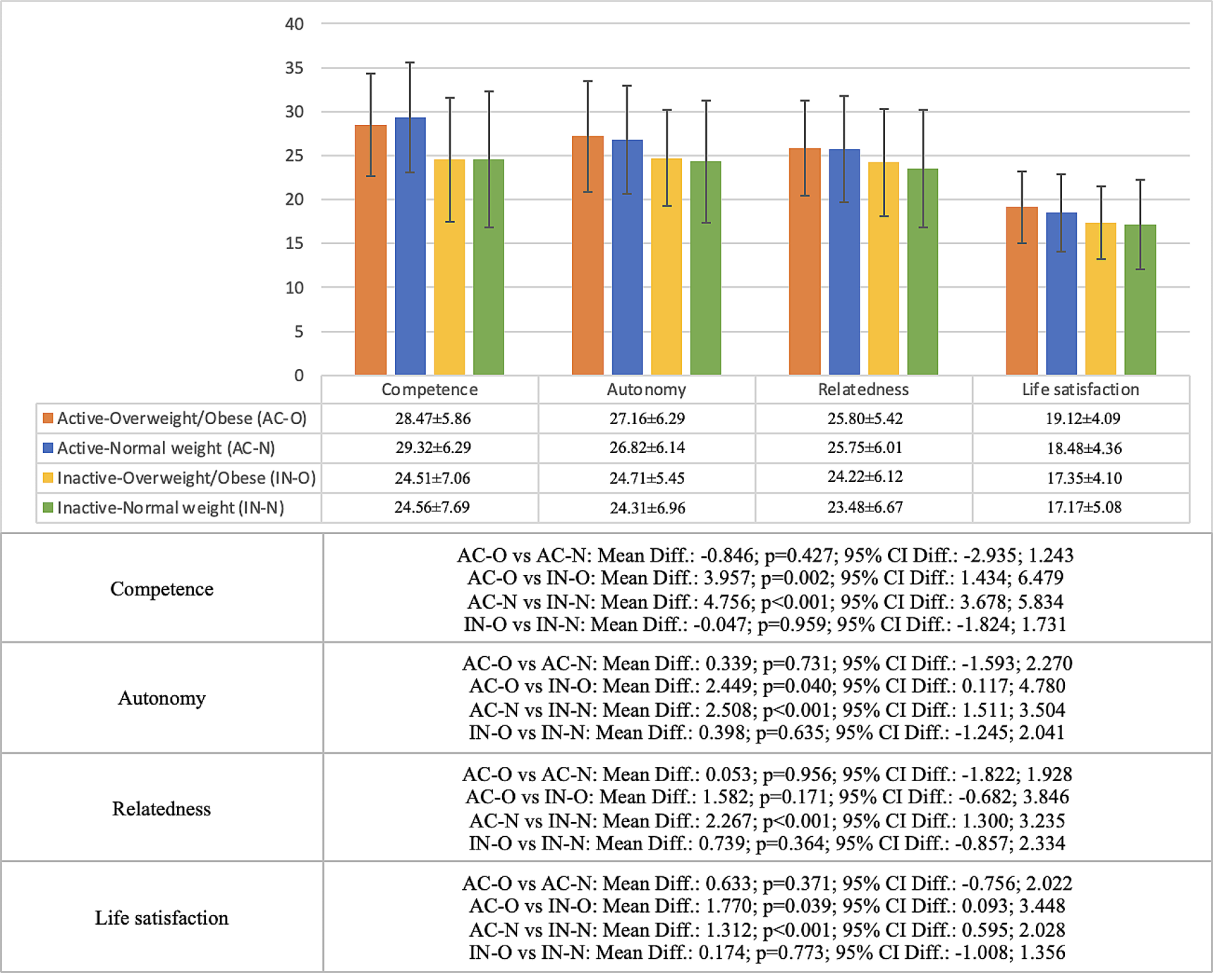
Differences in basic psychological needs (competence, autonomy, and relatedness) and life satisfaction among active and inactive adolescents with different weight status (normal weight or overweight/obese)

The differences in basic psychological needs and life satisfaction in active and inactive adolescents with normal weight and overweight/obesity are shown in Fig. 1. The results showed that in competence, autonomy, and life satisfaction there were significant differences between active and inactive adolescents with the same weight status. Thus, in both normal weight adolescents (competence, autonomy, and life satisfaction: *p* < 0.001) and overweight/obese adolescents (competence: *p* = 0.002; autonomy: *p* = 0.040; life satisfaction: *p* = 0.039), active adolescents had higher scores on these variables. In the case of relatedness, there was a higher score only in the active adolescents who were normal weight (*p* < 0.001), but not in the overweight/obese group (*p* = 0.171). There were no significant differences in any of the study variables when comparing normal weight adolescents with overweight/obese adolescents when they had the same level of physical activity (*p* = 0.364–0.959).

**Fig. 2:**
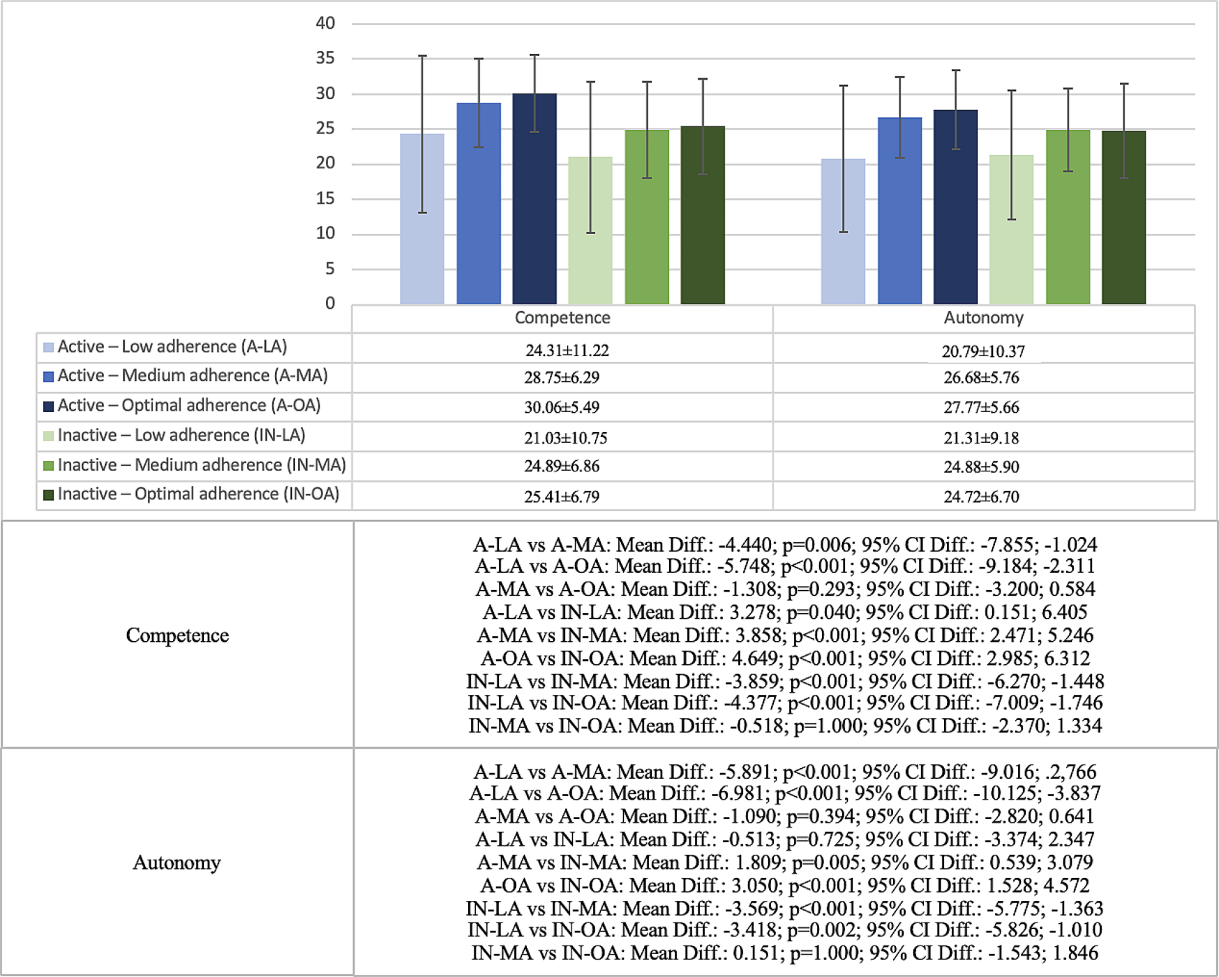
Differences in competence and autonomy among active and inactive adolescents with different adherence to Mediterranean diet (low adherence, medium adherence, or optimal adherence)

**Fig. 3:**
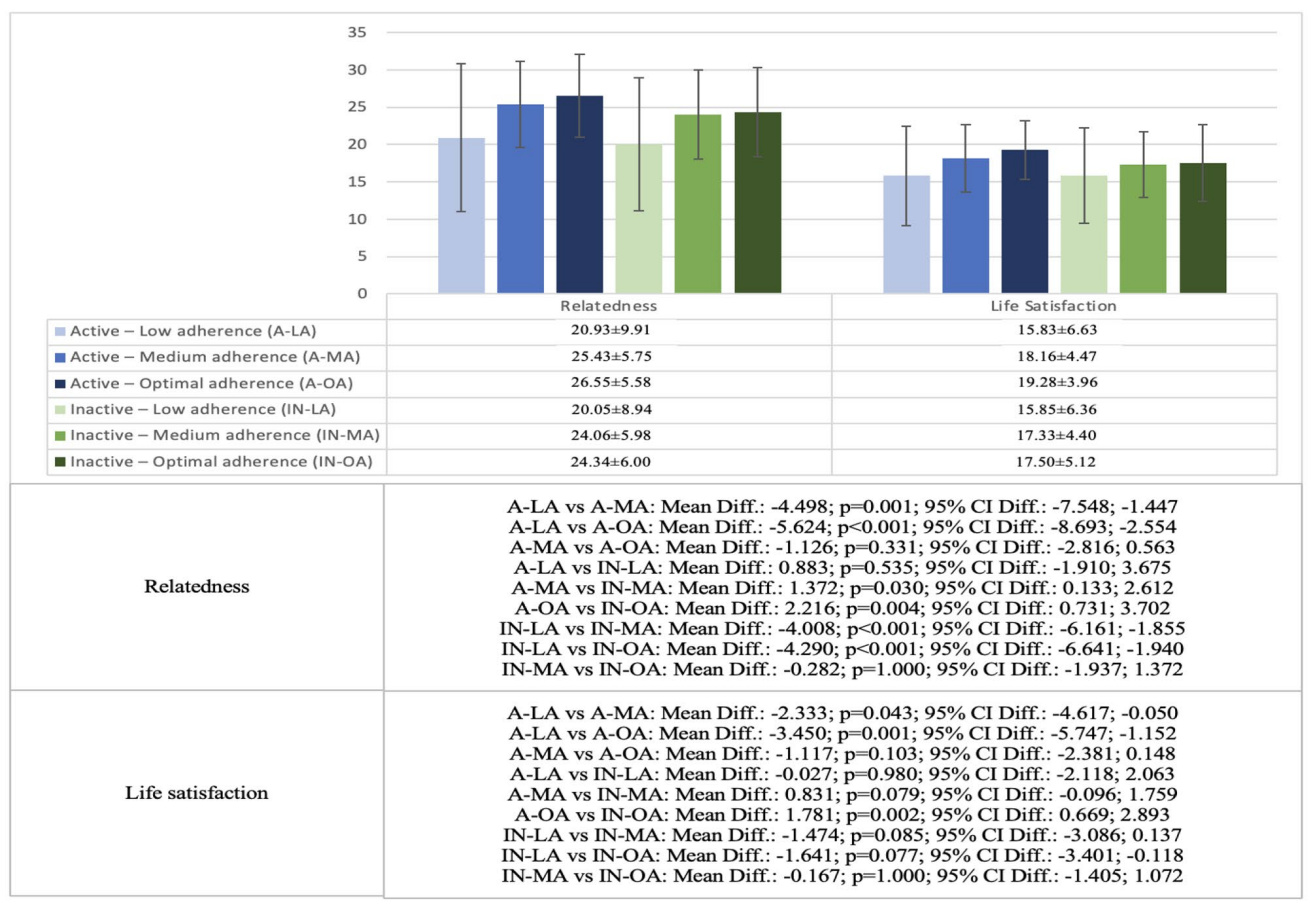
Differences in relatedness and life satisfaction among active and inactive adolescents with different adherence to Mediterranean diet (low adherence, medium adherence, or optimal adherence)

Figure 2 shows the differences in competence and autonomy; and Fig. 3 shows the differences in relatedness and life satisfaction in active and inactive adolescents with different levels of AMD. In the case of competence and autonomy, active adolescents with low adherence showed lower scores than active adolescents with medium (competence: *p* = 0.006; autonomy: *p* < 0.001) and optimal (competence and autonomy: *p* < 0.001) adherence. Similarly, inactive adolescents with low adherence showed lower scores on both variables than those with medium (competence and autonomy: *p* < 0.001) and optimal adherence (competence: *p* < 0.001; autonomy: *p* = 0.002). When comparing active and inactive adolescents with the same level of AMD, it was observed that active adolescents always showed higher scores in competence (low adherence: *p* = 0.040; medium adherence: *p* < 0.001; optimal adherence: *p* < 0.001), but not in autonomy, where differences were found only in adolescents with medium (*p* = 0.005) and optimal adherence (*p* < 0.001) (Fig. 2).

Similarly, active adolescents with low adherence showed lower scores in relatedness and life satisfaction than those with medium adherence (relatedness: *p* = 0.001; life satisfaction: *p* = 0.043) and optimal adherence (relatedness: *p* < 0.001; life satisfaction: *p* = 0.001). In the case of inactive adolescents, adolescents with low adherence showed lower scores than those with medium (*p* < 0.001) and optimal adherence (*p* < 0.001) in relatedness, but there were no significant differences in the case of life satisfaction (*p* = 0.077-1.000). And when comparing active and inactive adolescents with the same diet level, in the optimal adherence group, active adolescents showed higher scores in relatedness (*p* = 0.004) and life satisfaction (*p* = 0.002).

## Discussion

The first objective of the present study was to establish whether the differences between active and inactive adolescents in basic psychological needs and life satisfaction depend on gender. The results showed that active adolescents had higher scores than inactive adolescents on basic psychological needs and life satisfaction, with moderate effect sizes on all variables, but no differences when males and females were considered independently. Previous studies in this field found that the practice of physical activity generated an increase in life satisfaction, with significant differences for males [48]. A possible explanation for this would be that the systematic practice of physical activity is a determining factor in achieving and maintaining an adequate psychological state in the adolescent population, contributing to a greater psychological well-being at this age [49]. In addition, previous studies have shown that systematic physical exercise acts as a protective element against mental disorders such as depression, anxiety, or stress [50]. This could be due to the fact that, on the one hand, the practice of physical activity has a positive influence on the quality of life of adolescents mediated by the perception of self-concept and subjective happiness, with adolescents who practice more sports having a better quality of life and greater satisfaction [51]. On the other hand, the practice of sports could induce some changes at the hormonal level, highlighting the increase in serotonin production [52], with this hormone being widely associated with mental well-being due to its influence on the mood [53]. Although these results are interesting, the relationship between physical activity, basic psychological needs, and life satisfaction is mediated by other psychological variables, with self-esteem [54], hardiness and parental warmth having shown considerable influence in this regard [55]. Therefore, this is an interesting topic that needs to be expanded in future research.

The second objective of the present research was to determine the differences in basic psychological needs and life satisfaction of active and inactive adolescents with different weight status. Active adolescents in both normal weight and overweight/obese groups showed higher competence, autonomy, and life satisfaction, with moderate effect sizes in competence and autonomy, and small effect size in life satisfaction. These results are similar to those found in previous research in school-aged children, in which both normal weight and overweight/obese adolescents who took active breaks after sitting showed better mood and enjoyment than those who took sedentary breaks [56]. However, the results of the present research also differ from other research in obese adolescents, in which engaging in physical activity generated increased anxiety and fatigue [57]. Although future research is needed to obtain more information on this population, physical activity could be a protective factor for psychological well-being, even in overweight and obese populations, which have traditionally been associated with a worse psychological state [58]. Previous studies have shown that physical activity could decrease anxiety and increase self-steem in overweight/obese adolescents [59], and the results of the present research show benefits in competence, autonomy, and life satisfaction. These results give even more relevance to the practice of physical activity during childhood and adolescence, and it is therefore necessary that public and private institutions promote physical exercise due to the positive influence it seems to exert on different psychological variables [60, 61].

An important finding of the present research was that only active adolescents with normal weight showed a higher score in relatedness, with a moderate effect size; while in the overweight/obese group there were no significant differences between active and inactive adolescents in relatedness. This could be due to the fact that overweight/obese adolescents are teased and taunted by their peers during physical activity practice, and are also ignored, avoided, and excluded from physical activities [62], which would hinder the satisfaction of the relatedness need in this group, regardless of whether adolescents had higher levels of physical activity. This has been observed in previous research showing that weight-based victimization need not significantly decrease these adolescents’ physical activity practice, but peer teasing and bullying, humiliation, and feelings of insecurity about appearance are observed to be present during practice [63]. What is really worrying about this is that it ends up being a barrier to the practice of physical activity in this population, since these adolescents are victimized in school sports, out of school or both, greatly affecting their mental health [64]. In addition, if they finally drop-out the practice of physical activity, they are immersed in a circular process in which, in addition to the victimization suffered during sports practice [64], they are victimized for not meeting the physical activity guidelines [65], making them an extremely vulnerable population. This is an important aspect to consider, as physical activity during adolescence prevents up to 28% of the probability of becoming overweight or obese in adulthood [66]. As a solution to this situation, previous research has shown the effectiveness of designing specific social networks for overweight and obese adolescents through which young people communicate and feel equal to the rest of their peers, eliminating the negative perception of sports practice [67]. Also, the use of active video games in this population can be a useful resource to increase physical activity and motor skill development among these adolescents [68]. Future research is needed in this area to corroborate the effectiveness of these initiatives, but they could be a solution to the worrying situation found in the results of the present investigation.

Despite these results, no significant differences were found in any of the psychological variables studied when comparing active adolescents with different weight status or inactive adolescents with different weight status. These results would indicate that the level of physical activity could be the most determining factor in the differences found in the psychological variables of the study. However, no previous studies are known to have addressed psychological well-being from the joint perspective of physical activity and weight status, and in the case of the present study, the inclusion of overweight and obese adolescents as a single group could condition the results obtained. Therefore, although the results found are novel, the role of weight status in the differences found in the basic psychological needs and life satisfaction of active and inactive adolescents cannot be established, and future research is needed to further investigate this aspect. What can be indicated is that active adolescents score higher than inactive adolescents on these psychological variables, with the results giving even more relevance to the practice of physical activity.

The third objective of the present study was to analyse the differences in basic psychological needs and life satisfaction of active and inactive adolescents with different AMD. The results showed that adolescents with low AMD, regardless of whether they were active or inactive, presented lower satisfaction of basic psychological needs compared to adolescents with medium or optimal AMD, with moderate effect sizes for these findings. Previous research had shown that satisfaction of basic psychological needs was related to AMD, with adolescents with low adherence showing lower satisfaction [26], which is consistent with the results obtained in the present investigation. However, basic psychological needs and life satisfaction are not the only psychological variables that show differences when considering different levels of AMD, since previous studies have shown that adolescents with lower health-related quality of life or self-concept also had low AMD [23, 69, 70]. This is because self-concept and self-satisfaction are related to AMD [71, 72]. In this line, when adolescents have a low adherence to this nutritional pattern, these unhealthy nutritional habits may affect their body composition [73] and this factor is related to a poorer perception of their body image, reflected in a poorer self-concept and self-satisfaction [71, 72]. As there is a positive and significant relationship between adolescents’ basic psychological needs and self-concept and self-satisfaction [54], this could be the reason why adolescents with worse diets have lower scores in basic psychological needs. Future research including these variables is needed to corroborate the results obtained and to determine the role of basic psychological needs, self-satisfaction or self-concept in relation to the level of AMD in adolescents.

The present study is not free of limitations. It is important to note that the psychological variables were measured by means of questionnaires and, although they are valid and reliable, these instruments are limited in the field of psychology because they only collect part of the information. Although the role of physical activity and AMD has been considered, the possible impact of other variables such as socio-economic status, family environment or other lifestyle factors, such as sedentary activities or other psychological variables, have not been considered. Furthermore, in the division of the adolescents according to BMI, due to the small size of the samples, the overweight and obese adolescents had to be grouped together, so it was not possible to analyze the differences between these groups. In addition, when dividing the adolescents according to the level of AMD, the group of adolescents with a poor diet comprised a reduced sample, which could influence the subsequent statistical analyses; however, the groups could not be combined as in the case of BMI because the samples were even more disparate.

These limitations should be considered in future research in the field of adolescent mental health. Thus, based on the results obtained, it would be interesting that future studies analyze the differences in basic psychological needs and life satisfaction between active and inactive adolescents who are overweight or obese, using larger and more representative sample sizes, as in the present investigation, there did not seem to be differences between adolescents with the same level of physical activity and different weight status, but more information is needed to corroborate this. The fact that the effect sizes of the study were small/moderate is an aspect to be considered and makes consider the results cautiously, making future research necessary to provide more information in this regard. Furthermore, future research should determine which factors (level of physical activity, AMD, weight status) are statistically more influential on the basic psychological needs and life satisfaction of adolescents as this information may be very relevant for adolescent health promotion programs. Further research in this area is essential to prevent the development of mental illnesses, which unfortunately are increasingly appearing in children and adolescents due to poor nutritional patterns and physical habits [74]. Therefore, although the results found in the present research are interesting and novel, more follow-up studies are needed to support the findings.

## Conclusions

To conclude the present study, it should be noted that adolescent males and females who are active had higher values of basic psychological needs and life satisfaction than those who are inactive. The results follow the same line when considering the weight status of the adolescents, with active adolescents in the normal weight and overweight/obesity groups presenting higher values as compared to inactive adolescents with the same weight status. No significant differences were found when comparing normal weight and overweight/obese adolescents, regardless of their level of physical activity, but this information should be taken with caution since in this study overweight and obese adolescents were unified in the same group, preventing an accurate conclusion about the role of weight status in these relationships. And, with respect to AMD, it was found that adolescents with a similar level of physical activity, but lower AMD, showed significantly lower scores in basic psychological needs and life satisfaction as compared to their peers with a medium and optimal AMD. Therefore, based on the results obtained, the differences in the basic psychological needs and life satisfaction of adolescents are significant when considering the level of physical activity and AMD, but future studies are needed to know what happens with weight status.

## Data Availability

The data that support the findings of this study are available from the corresponding author, L. A-C., upon reasonable request.
